# Depletion of *Saccharomyces cerevisiae* in psoriasis patients, restored by Dimethylfumarate therapy (DMF)

**DOI:** 10.1371/journal.pone.0176955

**Published:** 2017-05-09

**Authors:** Hester Eppinga, H. Bing Thio, Marco W. J. Schreurs, Blerdi Blakaj, Ruena I. Tahitu, Sergey R. Konstantinov, Maikel P. Peppelenbosch, Gwenny M. Fuhler

**Affiliations:** 1 Department of Dermatology, Erasmus MC-University Medical Center, Rotterdam, the Netherlands; 2 Department of Gastroenterology and Hepatology, Erasmus MC-University Medical Center, Rotterdam, the Netherlands; 3 Department of Immunology, Erasmus MC- University Medical Center, Rotterdam, the Netherlands; Kermanshah University of Medical Sciences, ISLAMIC REPUBLIC OF IRAN

## Abstract

**Background:**

Psoriasis and inflammatory bowel disease (IBD) are chronic inflammatory diseases sharing similar pathogenic pathways. Intestinal microbial changes such as a decrease of bakers’ yeast *Saccharomyces cerevisiae* have been reported in IBD, suggesting the presence of a gut-skin axis.

**Objective:**

To investigate whether the *S*. *cerevisiae* abundance was altered in psoriasis patients versus healthy controls, and whether dimethylfumarate (DMF) interacted with this yeast.

**Methods:**

Using qPCR, faecal samples were compared between psoriasis patients without DMF (n = 30), psoriasis patients with DMF (n = 28), and healthy controls (n = 32).

**Results:**

Faecal *S*. *cerevisiae* abundance was decreased in psoriasis compared to healthy controls (p<0.001). Interestingly, DMF use raised *S*. *cerevisiae* levels (p<0.001). Gastrointestinal adverse-effects of DMF were correlated with a higher *S*. *cerevisiae* abundance (p = 0.010). *In vitro*, a direct effect of DMF on *S*. *cerevisiae* growth was observed. In addition, anti-*Saccharomyces cerevisiae* antibodies were not elevated in psoriasis.

**Conclusion:**

The abundance of baker’s yeast *S*. *cerevisiae* is decreased in psoriasis patients, but appears to be restored upon DMF use. *S*. *cerevisiae* is generally classified as a yeast with beneficial immunomodulatory properties, but may also be involved in the occurrence of DMF’s gastrointestinal adverse-effects. Potentially, DMF might be a new therapy for IBD.

## Introduction

Psoriasis and inflammatory bowel disease (IBD) are chronic inflammatory diseases sharing similar pathogenic pathways[1]. Both diseases are characterized by an increased inflammatory response at the epithelial barrier. Secondly, similar genetic susceptibility plays an important role in the development of both disease entities[2, 3]. A third contributing factor to IBD development is an altered intestinal microbial composition, with a decreased abundance of several commensal microbes (e.g. *Faecalibacterium prausnitzii*) and an increase of pathogens (e.g. adherent invasive *Escherichia coli*)[4]. Changes in the fungal microbial community are also common, with a decreased *Saccharomyces cerevisiae* abundance as one of the most prominent observations[5]. *S*. *cerevisiae*, also called “baker’s yeast” or “brewer’s yeast” because of its usage in fermentative production of bread, beer or wine, is the most intensively studied eukaryotic organism in literature and one of the most abundant yeasts in our gut. *S*. *cerevisiae* is known to possess anti-inflammatory properties by being able to stimulate IL-10 and inhibit TNF-α[5, 6]. Whether the fungal microbiome also plays a role in psoriasis is as yet unknown.

Dimethylfumarate (DMF) is an effective therapy for psoriasis, and an emerging therapy for multiple sclerosis (MS)[7, 8]. There is growing interest for the implementation of DMF for other chronic diseases such as IBD[9], although the exact mechanisms of action, besides immunomodulation, have not yet been fully elucidated[10]. DMF has been also applied as a biocide in shoe soles, clothes and furniture for prevention of mold growth. However, due to allergic eczematous reactions, its use in these applications has been discontinued[11, 12].

As IBD and psoriasis share similar pathogenic pathways, including similar bacterial disturbances in the gut as we have recently shown[13], we speculated that, as in IBD, *S*. *cerevisiae* abundance might be decreased in psoriasis. Interestingly, in Crohn’s disease, anti-*Saccharomyces cerevisiae* antibodies (ASCAs) are significantly elevated compared to healthy controls[14, 15]. Recently, elevated ASCA levels have also been demonstrated in patients with spondyloarthritis[16], but in psoriasis this has not been studied.

In this study we investigated whether psoriasis patients harbor an altered faecal *S*. *cerevisiae* abundance compared to healthy controls. Furthermore, in *vivo* and in *vitro*, we investigated whether DMF had impact on the *S*. *cerevisiae* abundance. In addition, ASCA levels were measured.

## Materials and methods

### Patients

All patients were included at the outpatient clinic of the Department of Dermatology, Erasmus MC in Rotterdam, the Netherlands. The study was approved by the medical ethical committee of the Erasmus MC (MEC-2014-371). Written informed consent was obtained for all participants. Inclusion criteria were a confirmed psoriasis diagnosis by a dermatologist, and age between 18–75 years. A minimum use of 6 weeks of DMF was required before inclusion in the study. Exclusion criteria were oral antibiotic use 8 weeks prior to inclusion, IBD-comorbidity, history of bowel resection, pregnancy and active infection. Clinical data was collected regarding medical history, comorbidities, medication, and disease characteristics. Duration of DMF use and presence of adverse-effects were recorded. Psoriasis Area and Severity Index (PASI) was used to assess disease activity (<10 mild, 10–20 moderate, >20 severe). From every participant, the faecal sample was sent by mail and stored at -80° Celsius within 48 hours.

A total of 49 psoriasis patients were included, of which the characteristics are depicted in Table 1. In total 30 samples were collected from psoriasis patients without DMF. A total of 28 samples were collected from psoriasis patients with DMF. Of nine of these patients, two samples were collected, one sample before and one sample after (6–9 weeks) start of DMF.

**Table 1:** Patients’ characteristics.

	Psoriasis - DMF	Psoriasis + DMF	Healthy controls	P-value^1^
**N** (patients)	30	28	32	
**Age** (mean yrs, SD)	46.1 (13.9)	42.7 (14.1)	42.6 (14.1)	P = 0.54
**Gender** (% female)	60.0	50.0	62.5	P = 0.52
**Smoking** (%)	20.0	28.6	6.3	P = 0.07
**BMI** (mean, SD)	27.8 (5.3)	27.2 (4.5)	25.3 (4.8)	P = 0.11
**Caucasian** (%)	80.0	82.1	81.3	P = 0.98
**Age at diagnosis** (mean yrs, SD)	30.8 (12.2)	25.4 (11.8)	NA	P = 0.09
**Disease duration** (mean yrs, SD)	15.7 (11.6)	17.0 (11.1)	NA	P = 0.67
**Psoriasis** type n (%)				
Vulgaris	25 (83.3)	25 (89.3)	NA	
Guttate	3 (10.0)	2 (7.1)	NA	
Palmoplantaris	2 (6.7)	1 (3.6)	NA	
**Psoriasis therapy**^2^				
Immunosuppressant	2	1	NA	
Local therapy	15	14	NA	
UVB therapy	2	0	NA	
**Duration DMF** (wks, mean, SD)	NA	66.8 (94.0)	NA	
**PASI**^3^				
< 10	16 (64.0)	20 (80.0)	NA	
>10 en ≤ 20	8 (32.0)	5 (20.0)	NA	
>20	1 (5.0)	0 (0)	NA	

DMF, dimethylfumarate; BMI, body mass index; NA, non-applicable

^1^ P-value calculated by one-way ANOVA for age, BMI; unpaired-t-test for age at diagnosis, disease duration; chi-square test for gender, smoking, ethnicity

^2^ Patient could have been on concomitant drugs

^3^ PASI, Psoriasis Area Severity Index for plaque type at time of sample collection (<10 mild, 10–20 moderate, >20 severe). Note that 9 of the 28 patients on DMF are paired with the no-DMF group.

### DNA isolation and quantitative polymerase chain reaction (qPCR)

DNA was extracted from 20 mg faeces per sample as described previously[13]. Briefly, 1 ml of cell lysis buffer (Ambion, Life Technologies) was added, followed by 15 min incubation period. Full cell lysis was achieved by bead-beating [three times 30s]. The samples were centrifuged, whereupon 3:1 protein precipitation buffer (Promega) was added; 100% isopropanol [1:1] was used to precipitate DNA from the supernatant; 100μl 70% ethanol was used to wash the DNA pellet. Finally, DNA was resuspended in 50μl TE-buffer, DNA concentration was measured on a Nanodrop spectrophotometer [Isogen Life Science BV, De Meern, The Netherlands] and diluted to 10 ng/μl.

QPCR was performed two times as described[13]. QPCRs were performed in duplicate for *S*. *cerevisiae* using the following primer:

F-AGGAGTGCGGTTCTTTG; R-TACTTACCGAGGCAAGCTACA[5].

Bacterial abundance analysis was performed by SybrGreen based qPCR reaction containing 20ng DNA [2**μ**l of 10ng/**μ**l], 9**μ**L SYBR®Select MasterMix for CFX (ThermoFisher Scientific), 7μl dH20, 1μl 10μM forward primer, and 1μl 10μM reverse primer.

Thermocycle conditions comprised: denaturation step 10 min at 95°C; 40 cycles of 95°C denaturation for 15s; 56°C primer annealing for 30s; and 72°C extension for 30s followed by a standard melting

curve analysis. The mean was calculated from the two qPCRs and the *S*. *cerevisiae* abundance was expressed in log10copies/gram faeces.

### XTT assay

In vitro analysis was performed to establish whether the change in *S*. *cerevisiae* abundance was directly or indirectly induced by DMF. The yeast *S*. *cerevisiae* (MycoBank#:163963) was obtained from CBS-KNAW Fungal Biodiversity Centre, institute of the Royal Netherlands Academy of Arts and Sciences. The yeast was cultured at 30°C for 48 hours and 100μl of a 0.1McFarland yeast suspension was pipetted into a 96-well plate. Serial dilutions of DMF, fumaric acid and an antimycotic (amphotericine-B-deoxycholate) were added to the cells in triplicate. H_2_0 and ethanol were used as vehicle control. XTT/PMS was added to the suspension as per manufacturers’ directions (Molecular Probes/Thermo Scientific) and the plate was read after 24h at OD415 nm. Six independent experiments were performed.

### ASCA measurement

Serum was collected of 30 psoriasis patients, including 18 without DMF and 12 with DMF, and 17 healthy controls, for the determination of anti-*Saccharomyces cerevisiae* antibodies (ASCAs), IgA and IgG. Results were categorized as following: ASCAs (units) <20 negative; 20–25 borderline positive; >25 positive. ASCAs were determined using QUANTALite ASCA IgA/IgG ELISA (Inova Diagnostics, San Diego, CA). ELISA was performed according to the manufacturer’s instructions, without modifications.

### Statistical analysis

Patients’ characteristics were compared between psoriasis patients without DMF use, psoriasis patients with DMF use, and healthy controls by using the one-way analysis of variance (ANOVA), unpaired t-test and chi-square test, depending of the presence of a normal distribution. IBM SPSS 21.0 statistical software, Armonk, NY, USA was used for the statistical analyses. *In vitro* data were analyzed using Graphpad Prism (version 5.1), performing paired-t-tests.

## Results

### Patients’ characteristics

There were no significant clinical differences between the groups (Table 1). Nine participants (10%) followed a specific diet. In the psoriasis patients without DMF, one followed a sugar-free and one a cow-milk- and wheat-free diet. In the psoriasis patients with DMF, one followed a gluten-free, one a low-carbohydrate, one a both vegetarian and low-carbohydrate diet and one a dairy-free diet. Of the healthy controls, three were vegetarian. In the electronic charts, none of the included patients were reported to have used oral antifungal or antibiotic medication 8 weeks before the study or during the study. In the psoriasis patients without DMF, the majority had plaques on multiple body sites (diffuse) (n = 28, 88%). Two had solely plaques on their feet, and two solely on their head. In the psoriasis patients with DMF, plaques were located diffusely in 26 patients (93%). One had solely psoriasis plaques on the neck and scalp, and one on the feet.

### Significant reduction of *S*. *cerevisiae* abundance in psoriasis, corrected by DMF treatment

**Fig 1:**
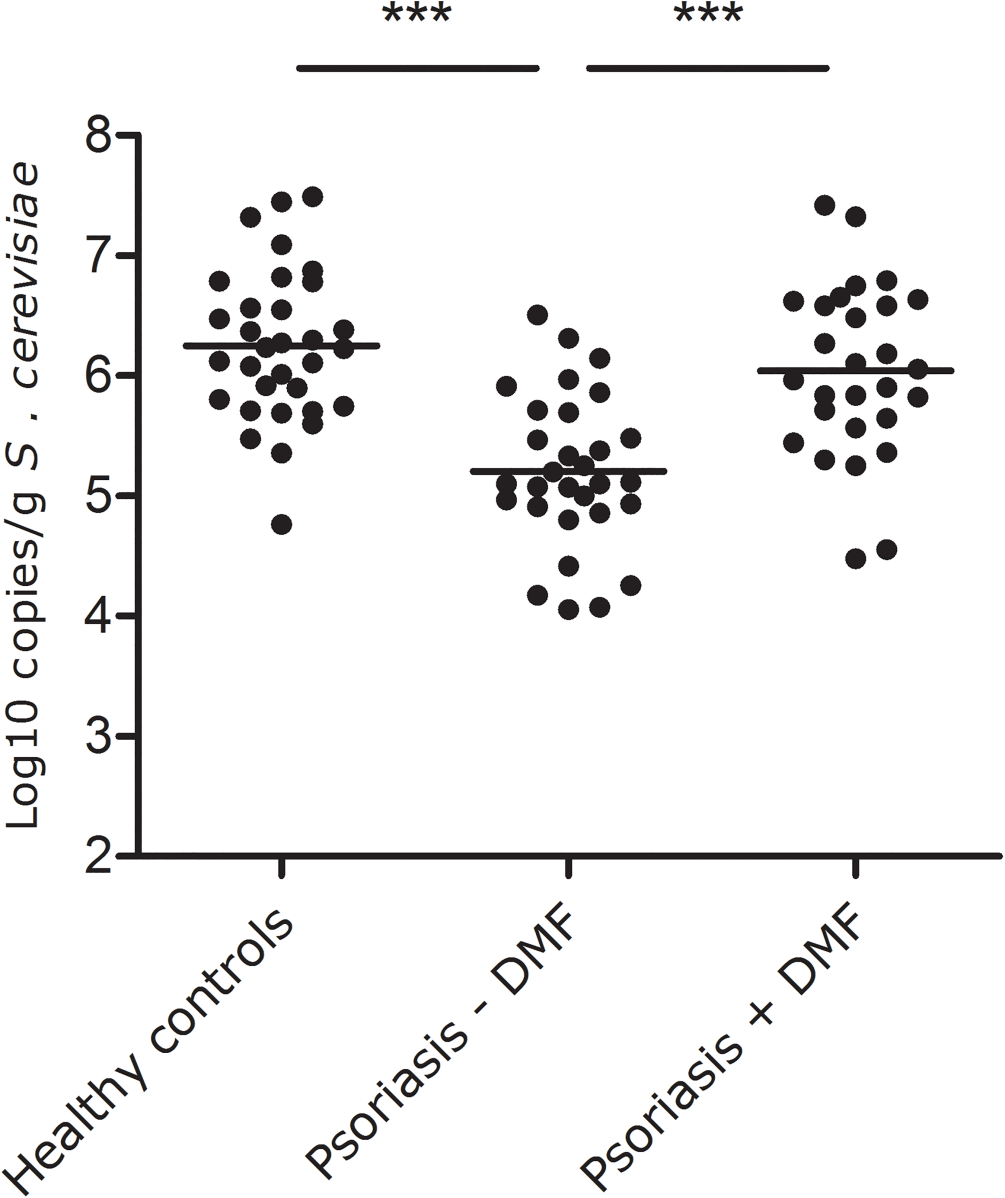
Psoriasis patients without DMF had a significantly lower faecal *Saccharomyces cerevisiae* abundance than healthy controls and psoriasis patients on DMF (both p<0.001). Psoriasis patients using DMF had similar *S*. *cerevisiae* abundance compared to healthy controls (p = 0.233). The middle line represents the average abundance.

A significant difference in *S*. *cerevisiae* abundance was demonstrated by one-way ANOVA of the *S*. *cerevisiae* abundance between the three groups (1. psoriasis patients without DMF, 2. psoriasis patients with DMF, 3. healthy controls; p<0.001). Psoriasis patients without DMF had a significantly lower *S*. *cerevisiae* abundance than healthy controls (mean log10 copies/g± SD: 5.20±0.64 vs 6.25±0.63, p<0.001). Psoriasis patients using DMF had a significant higher *S*. *cerevisiae* abundance than patients without DMF (6.04±0.72, p<0.001), reaching levels that were similar to those of healthy controls (p = 0.233, Fig 1). Excluding the psoriasis patients using systemic immunosuppressant’s (n = 2 without DMF, n = 1 with DMF) did not affect the results. A subgroup analysis was performed for the patients for whom paired samples were available, before and after DMF (n = 9). Within this group there was also a trend observed towards an increased *S*. *cerevisiae* abundance upon DMF treatment (before DMF, 4.99±0.64; after DMF 5.62±0.60; p = 0.086). All patients showed clinical response to DMF, and PASI scores did not correlate to the *S*. *cerevisiae* abundance.

### The presence of side-effects of DMF correlate with a higher *S*. *cerevisiae* abundance

**Fig 2:**
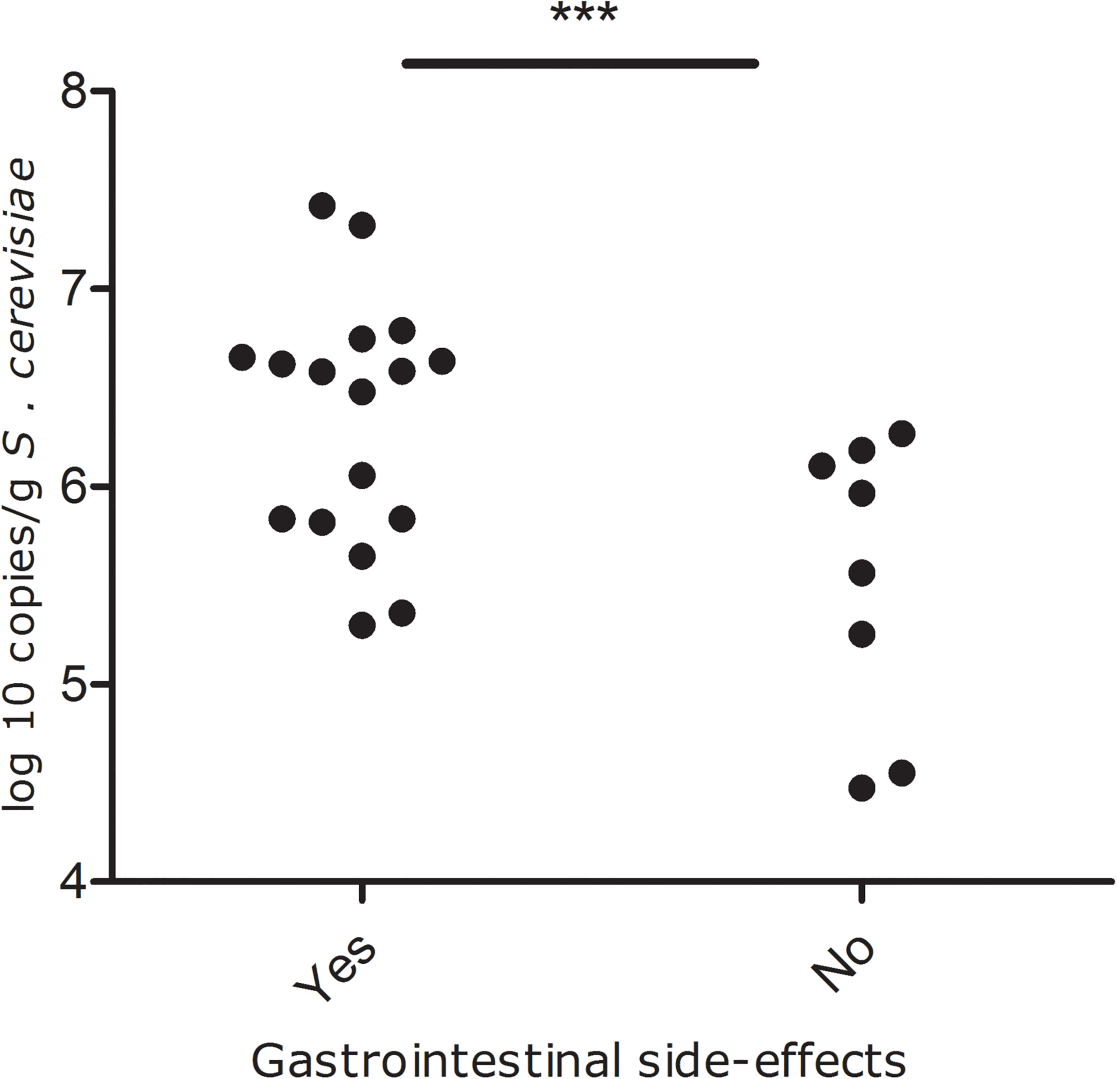
Psoriasis patients on DMF who had gastrointestinal side-effects had a significantly higher *S*. *cerevisiae* abundance than psoriasis patients without the side-effects (p = 0.010).

The majority (20 of 25 patients (80%), n = 3 missing) of the psoriasis patients who used DMF reported adverse effects of DMF use. The most reported adverse effects were flushing (42.9%), diarrhea (32.1%), abdominal pain (25.0%) and nausea (14.3%). Gastrointestinal side-effects (abdominal pain, diarrhea and/or nausea), were reported in 17 patients on DMF, while 8 patients had no side-effects. Patients with gastrointestinal side-effects had a significant higher *S*. *cerevisiae* abundance than patients without these side-effects (6.33±0.63 vs 5.55±0.72; p = 0.010, Fig 2). When we included all adverse-effects, including flushing, similar results were observed (6.26±0.63 vs 5.37±0.81; p = 0.013).

### DMF directly stimulates *S*. *cerevisiae* growth *in vitro*

**Fig 3:**
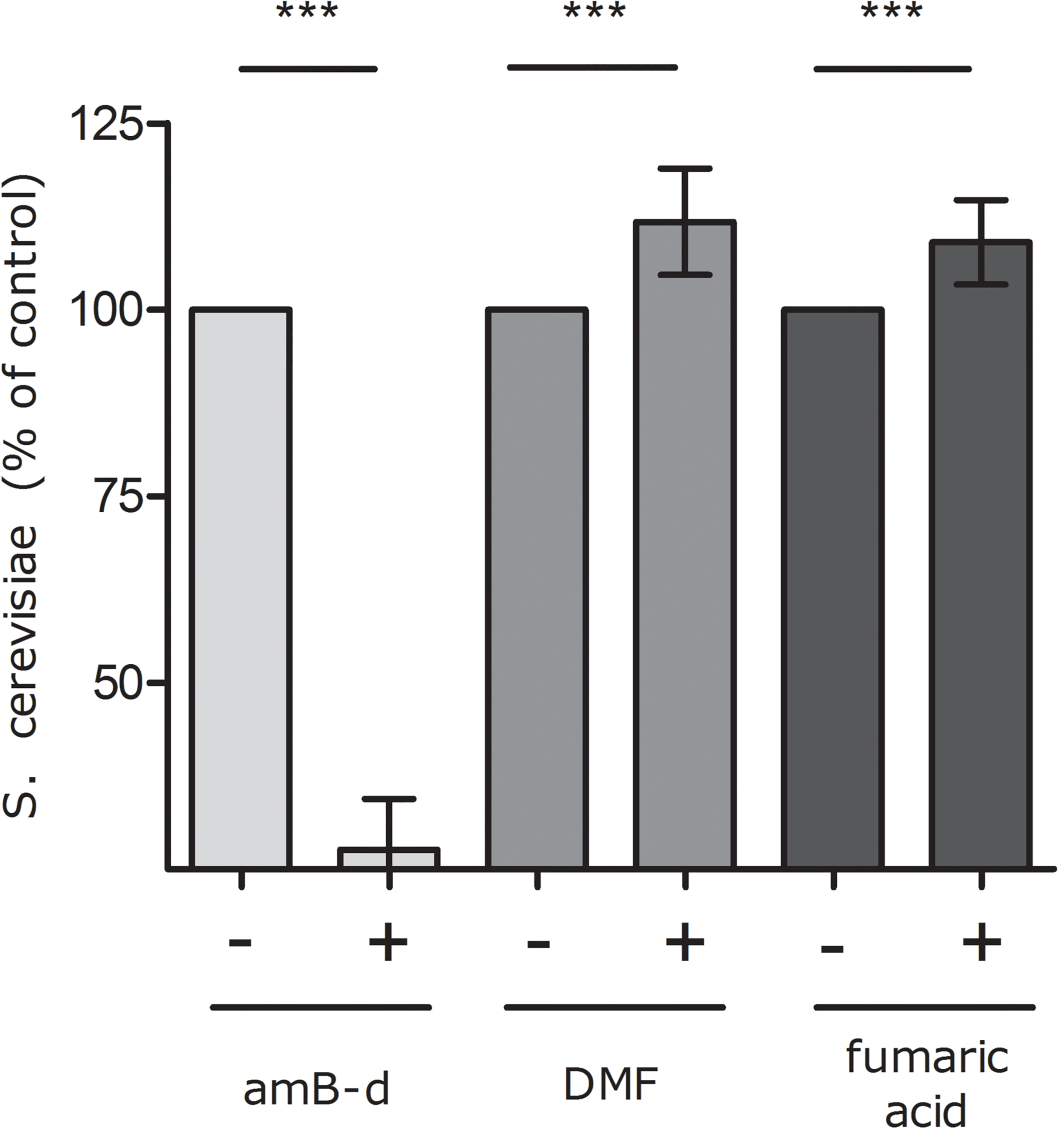
DMF (1.6 mg/ml) and fumaric acid (0.46 mg/ml) significantly stimulate *S*. *cerevisiae* growth (p = 0.01), amphotericine B-deoxycholate (amB-d) inhibits *S*. *cerevisiae*as expected. Maximal concentration fumaric acid used (0.46 mg/ml) was limited by the dissolvent, ethanol. Data are expressed as mean, SD.

To investigate whether the increase in *S*. *cerevisiae* in faeces from patients treated with DMF was a direct effect of DMF on the yeast itself, we treated S. cerevisiae *in vitro* with DMF. Results show that DMF significantly induces *S*. *cerevisiae* growth after 24 hours in comparison to untreated cells (p = 0.0128; Fig 3). DMF is a dimethyl ester of fumaric acid, and addition of fumaric acid directly to *S*.*cerevisiae* also resulted in a significant increase in yeast growth (p = 0.010). Administration of the antimycotic amphotericine-B-deoxycholate resulted, as expected, in a significant decrease of cultured *S*. *cerevisiae* (p<0.001). In addition, to exclude the possibility that the depletion of *S*. *cerevisiae* in psoriasis was due to lower faecal fumaric acid levels in these patients, fumaric acid measurement in faeces was performed in 16 paired psoriasis samples, without DMF use and with DMF, and in 8 healthy controls. No significant differences in fumaric acid levels between these groups were found (see supplementary data, in particular S2 Fig).

### Anti-*Saccharomyces cerevisiae* antibodies (ASCAs)

The ASCA IgA level was elevated in one (3.3%) of the 30 psoriasis patients. This patient used DMF. Two patients (6.7%) (both not on DMF) had a borderline positive ASCA IgA, compared to one (5.9%) healthy subject (total n = 17).

The ASCA IgG level was elevated in 3 out of 30 patients (10%), of which two with and one without DMF. One of them had also a positive IgA level. Of the healthy subjects two (11.8%) had a positive IgG ASCA, which were different subjects than the healthy control with a positive IgA. No correlation between a positive ASCA and faecal *S*. *cerevisiae* abundance was found.

## Discussion

This study demonstrates a significant depletion of *S*. *cerevisiae* in psoriasis, which appears to be restored in psoriasis patients on DMF. Additionally, our study confirmed that, *in vitro*, DMF can directly stimulate *S*. *cerevisiae* growth.

To date, information on the impact of medication on the (bacterial or fungal) microbiome is still scarce, and this study shows we should not underestimate the consequences of medication use, whether they are beneficial or harmful to our microbiome. It is unclear whether microbes and medication act synergistically or whether microbial changes could be a result of the disease remission and the anti-inflammatory milieu caused by the medication. Our *in vitro* data showing that DMF directly stimulates the growth of *S*. *cerevisiae* suggests that the increased colonisation is not only the result of an anti-inflammatory environment. Recent evidence shows that *S*. *cerevisiae* itself exhibits several immunomodulatory properties. The cell wall of *S*. *cerevisiae* consists mainly of β-glucans, which has immunomodulatory effects[17], and TNF-α reduction and IL10 stimulation by *S*. *cerevisiae* have recently been shown[5, 6]. TNF-α (pro-inflammatory) and IL10 (anti-inflammatory) are important cytokines in the pathogenesis of chronic inflammatory immune-mediated diseases such as psoriasis and IBD. Thus, a higher *S*. *cerevisiae* abundance might contribute to the anti-inflammatory effect of DMF. In addition to the immunomodulatory effects of DMF itself, a DMF-induced increase of *S*. *cerevisiae* might contribute to achievement (and maintenance) of stable remission in patients, providing a beneficial milieu for colonization. The finding that DMF seems to restore a (fungal) disturbance in psoriasis, could also be extrapolated to the *S*. *cerevisiae* depletion observed in IBD[5, 9], or potentially, MS. Psoriasis and IBD share common pathophysiological processes, including common genetic risk factors [18, 19]. Interestingly, it was recently demonstrated that in IBD, a risk-associated single nucleotide polymorphism (SNP) in the CARD9 gene negatively correlates with *S*. *cerevisiae* abundance, suggesting that genetic variants affecting immune cell function can modulate microbial abundance [5]. However, to the best of our knowledge, the CARD9 IBD-risk allele has so far not been associated with psoriasis [19, 20]. Furthermore, while SNPs in other immunity genes may potentially contribute to an altered microbial balance, it is important to note that such disease-associated alleles, while more prevalent in disease, are also present in healthy individuals, and are therefore unlikely to (solely) explain the decreased *S*. *cerevisiae* abundance in the psoriasis cohort as compared to healthy controls.

*S*. *cerevisiae* is one of the most abundant members of the fungal microbiota and might exert favorable immunologic effects in immunological diseases. However, it is important to note that from a greater perspective DMF potentially also interacts with other members of the fungal and/or bacterial microbiome. Thus, while our *in vitro* data show a direct effect of DMF on *S*. *cerevisiae* growth, it is also conceivable that other microbes might be able to influence *S*. *cerevisiae* and vice versa. Therefore more research into the other members of the microbiome is warranted.

It is of interest to note that the benefit of *S*. *cerevisiae* when used as skin-conditioning agent has been demonstrated earlier[21]. Our study outlines the potential beneficial role of *S*. *cerevisiae* in skin (and gut) homeostasis. At present, *S*. *cerevisiae* is also used as dietary supplement because of its nutrients (rich in amino acids) and functions. It is unknown whether *S*. *cerevisiae* supplemented in food colonizes the intestine similarly as the stimulation of (probably resident) *S*. *cerevisiae* by DMF. Some of the patients included in our study followed a particular diet which might potentially impact the response of DMF, its side-effects and/or the *S*. *cerevisiae* abundance, although we did not observe such trend. Interestingly, a variant of *S*. *cerevisiae*, *Saccharomyces boulardii*, is already used as probiotic for diarrhea. Besides *S*. *cerevisiae*, other members of the fungal microbiome could be of interest, and moreover, bacteria could be influenced by DMF as well.

However, while the potential use of *S*. *cerevisiae* (and DMF) as probiotic is interesting, it should, in the light of our study, be carefully implemented after further investigations into its effect and safety[22]. Our study showed that a higher *S*. *cerevisiae* abundance might also have less favorable effects: the presence of gastrointestinal side-effects, such as diarrhea, nausea and abdominal pain, which are often reported side-effects of DMF therapy, were correlated with a significant higher *S*. *cerevisiae* abundance. Nevertheless, although in the past decades an increasing incidence of (opportunistic) infections involving *S*. *cerevisiae* have been described[23], *S*. *cerevisiae*, which is used in baking and brewing, is still considered to be safe and non-pathogenic.

A strength of this patient cohort was that 93% did not use additional systemic medication, excluding other medication bias. Future studies should include a complete paired-sample set, and also investigate other members of the fungal microbiome in psoriasis.

In conclusion, *S*. *cerevisiae* is depleted in psoriasis patients. In *in vitro* and *in vivo* experiments, DMF is able to stimulate the growth of *S*. *cerevisiae*, which might play a role in the etiopathogenesis of psoriasis and possible also other diseases such as MS and IBD. It is also conceivable that *S*. *cerevisiae* acts as a bystander, however not innocently, as it might be the cause the gastrointestinal adverse-effects of DMF. Further investigations should look into the immunologic and therapeutic functions of *S*. *cerevisiae* and the mechanistic effect of DMF and other medications on the inhabitants of our gut. *S*. *cerevisiae* as probiotic might be a potential candidate for novel treatments in patients with chronic inflammatory diseases such as psoriasis.

## Supporting information

S1 Text Supplementary text. (DOCX)

S1 Fig (TIF)

S2 Fig (TIF)
