# Supplementary material for: Depletion of Saccharomyces cerevisiae in psoriasis patients, restored by Dimethylfumarate therapy (DMF)
Source: PLoS One. 2017 May 9;12(5):e0176955. doi: 10.1371/journal.pone.0176955 (PMC5423625; doi:10.1371/journal.pone.0176955)
Supplement: S1 Text — (DOCX) [file pone.0176955.s001.docx]

**Supplementary Text**

# Faecal fumaric acid measurement

# A Fumarate Detection Kit (Abcam 102516) was used for fumaric acid measurement in faeces. Forty mg of faeces was weighed, and 100 μl Assay Buffer was added to the samples. Bead-beating (2 times 30 seconds) was performed for homogenization of the samples, followed by centrifugation for 10 minutes (13.000 rpm). The protein concentration of the supernatant was measured with the DC protein quantification kit Bradford (Hercules, CA). To the supernatant of the faeces and the Assay buffer (total 50 μl), 100 μl reaction mix was added, which consisted of 90 μl Fumarate Assay Buffer, 8 μl Fumarate Developer and 2 μl Fumarate enzyme mix per sample. This was followed by 60 minutes of incubation at 37°C. The absorbance was measured at 450 nm in a microplate reader. OD measurements of samples were converted to μg/ml fumaric acid by means of standard curves included in each assay.

# Psoriasis patients show normal levels of fumaric acid in faeces

# As *in vitro* both fumaric acid and its ester can stimulate growth of *S. cerevisiae*, and abundance of this organism was decreased in stool from psoriasis patients, we next wondered whether fumaric acid levels in stool from psoriasis patients were affected (and would be lower than normal). We used a commercial kit, which detects only fumaric acid and not its esters (this was tested in the assay). As fumaric acid measurement in stool using this assay has not been described before, we first performed a pilot experiment on stool samples from three individual donors, showing variable amounts of fumaric acid (Supplementary Fig 1) in these samples, using a range of faecal sample input. Supplementary Fig 1 demonstrates that differences of fumaric acid in individual stool samples can be quantitatively measured using this assay.

# We subsequently measured the presence of fumaric acid in 8 faecal samples of psoriasis patients without DMF and in 8 faecal samples of psoriasis patients on DMF treatment, as well as in faecal samples of 8 healthy controls. A wide variety in fumaric acid levels in stool was observed, but no significant differences between the groups were demonstrated (Supplementary Fig 2).

# S1 Fig. Different concentrations of faecal sample demonstrate different levels of fumaric acid (OD) in three (test) subjects.

# S2 Fig. Measurement of faecal fumaric acid levels did not demonstrate a significant difference between the groups (p=0.540) (mean, SD).
