# Supplementary figures and images for: Depletion of Saccharomyces cerevisiae in psoriasis patients, restored by Dimethylfumarate therapy (DMF)
Source: PLoS One. 2017 May 9;12(5):e0176955. doi: 10.1371/journal.pone.0176955 (PMC5423625; doi:10.1371/journal.pone.0176955)

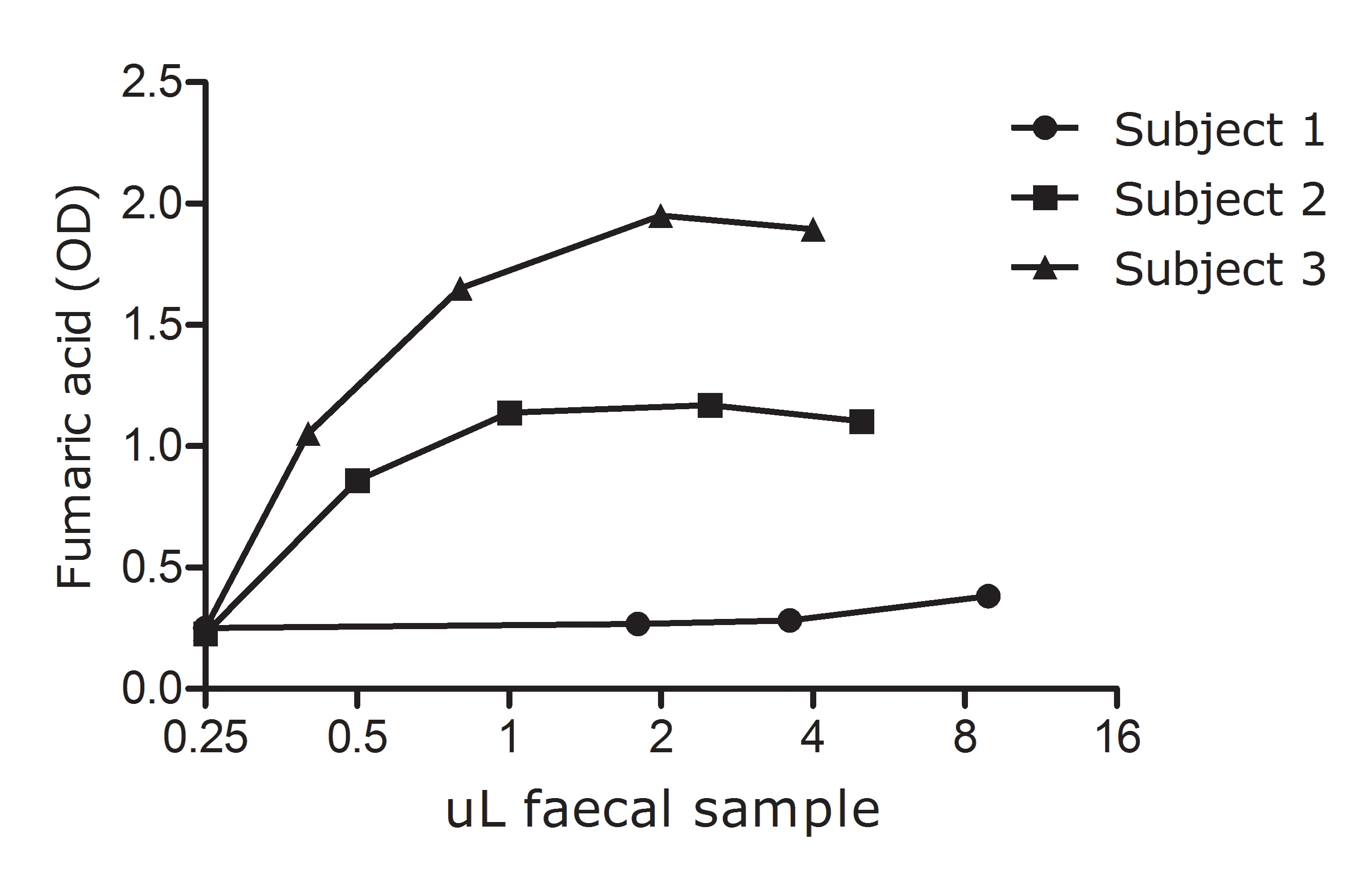

Supplement: S1 Fig — (TIF) [file pone.0176955.s002.tif]

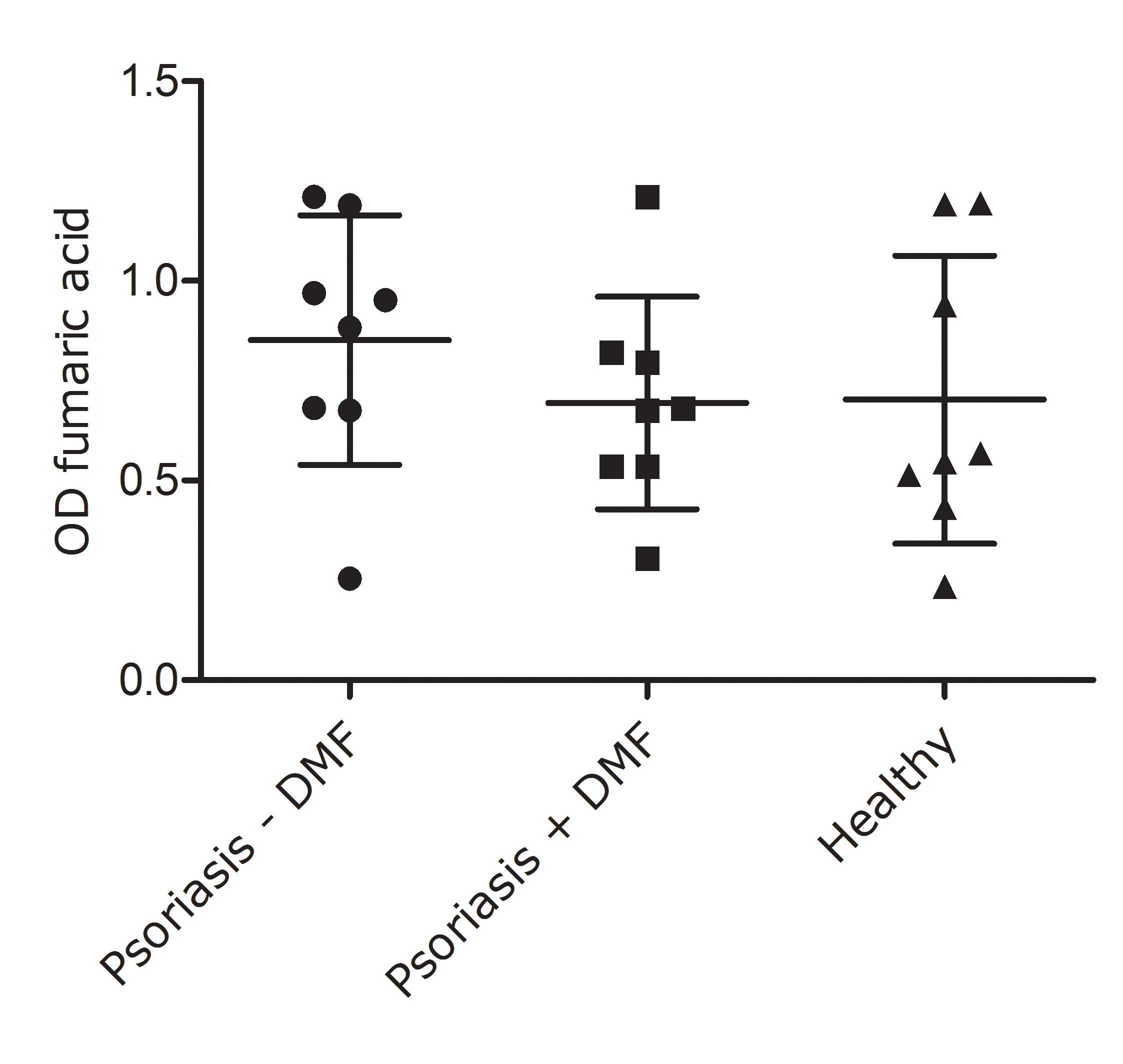

Supplement: S2 Fig — (TIF) [file pone.0176955.s003.tif]
